# 

**Published:** 1863

**Authors:** 


### CONTENTS.

### ORIGINAL CONTRIBUTIONS.

ART. VIII. A Case of Amputation of the Femur, for Mortificaton of the Leg, with Two Cases Catalytic Disease, Treated with the Sulphites of Soda and Lime. By A. Fisher, M.D., of Chicago,... 129
ART. IX. The Selection of Remedies, and the Art of Prescribing Them. By Swayne Wickersham, M.D., of Chicago,.................. 139
ART. X. Creosote in Hospital Gangrene. By C. R. Parke, M.D., Bloomington, Ill.,........................................ 149
ART. XI. Deaf Mute Restored to Hearing by an Attack of Variola. By J. J. Samuels, Marion, Ill.,................................ 151

### THE CLINIQUE.

Surgical Clinic on Injuries of the Elbow-Joint. By Prof. E. Andrews, 157
Medical Wards of the Mercy Hospital. Erysipelas,—Sulphite of Lime, &c. By N. S. Davis, M.D., Professor of Practical and Clinical Medicine, &c.,............................................ 161

### PROCEEDINGS OF SOCIETIES.

Army Medical Society, at Jackson, Tenn.,................................ 166

### BOOK NOTICES.

The Principles and Practice of Surgery. By Henry H. Smith, M.D., Prof. of Surgery in the University of Pennsylvania, &c........................ 168
Transactions of the Ohio State Medical Society, 1862,........................ 169
Report of the Board of Health of the City of Philadelphia for 1862. By James A. McCrea, M.D., and William Read, Esq........................ 169
Braithwaite's Retrospect of Practical Medicine and Surgery,................ 169
London Lancet,........................................................ 170

### SELECTIONS.

Insanity and Intemperance. By Andrew McFarland, M.D.,............... 170

### EDITORIAL.

Illinois State Medical Society,—Annual Meeting,............................ 190
Illinois State Medical Society, ............................................ 200
Explanation, .............................................................. 201
Characteristic, ........................................................... 201
Calomel and Tartar Emetic in the Army, ..................................... 201
Cure of Bright's Disease by a Milk Diet, ................................... 202
Death of Dr. Charles Hooker,................................................ 204
Death of Dr. Charles Fishback, ............................................. 205
Obituary Record, ........................................................... 205
Resignation of Professor H. H. Childs,...................................... 206
Washington City, ........................................................... 206
Sulphate of Morphia Administered through the Ear,........................... 207
How they Live in New York,.................................................. 207

